# *POLRMT* as a Novel Susceptibility Gene for Cardiotoxicity in Epirubicin Treatment of Breast Cancer Patients

**DOI:** 10.3390/pharmaceutics13111942

**Published:** 2021-11-16

**Authors:** Alejandro Velasco-Ruiz, Rocio Nuñez-Torres, Guillermo Pita, Hans Wildiers, Diether Lambrechts, Sigrid Hatse, Danielle Delombaerde, Thomas Van Brussel, M. Rosario Alonso, Nuria Alvarez, Belen Herraez, Christof Vulsteke, Pilar Zamora, Teresa Lopez-Fernandez, Anna Gonzalez-Neira

**Affiliations:** 1 Human Genotyping Unit, CeGen (Spanish National Genotyping Centre), Human Cancer Genetics Programme, Spanish National Cancer Research Centre (CNIO), Calle de Melchor Fernández Alamagro, 3, 28029 Madrid, Spain; avelascor@cnio.es (A.V.-R.); rnunez@cnio.es (R.N.-T.); gpita@cnio.es (G.P.); mralonso@cnio.es (M.R.A.); nalvarez@cnio.es (N.A.); bherraez@cnio.es (B.H.); 2 Department of General Medical Oncology, University Hospital of Leuven, Herestraat 49, 3000 Leuven, Belgium; hans.wildiers@uzleuven.be; 3 Multidisciplinary Breast Centre, University Hospital of Leuven, Herestraat 49, 3000 Leuven, Belgium; sigrid.hatse@kuleuven.be; 4 Laboratory of Experimental Oncology (LEO), Department of Oncology, Katholieke Universiteit (KU) Leuven, Oude Markt 13, 3000 Leuven, Belgium; 5 Laboratory of Translational Genetics, Centre for Cancer Biology (CCB), Flanders Institute for Biotechnology (VIB), Rijvisschestraat 120, 9052 Leuven, Belgium; diether.lambrechts@vib-kuleuven.be (D.L.); thomas.vanbrussel@vib-kuleuven.be (T.V.B.); 6 Integrated Cancer Center Ghent, Department of Medical Oncology, AZ Maria Middelares, 9000 Ghent, Belgium; danielle.delombaerde@azmmsj.be (D.D.); christof.vulsteke@azmmsj.be (C.V.); 7 Center for Oncological Research (CORE), Integrated Personalized and Precision Oncology Network (IPPON), University of Antwerp, 2610 Wilrijk, Belgium; 8 Department of Medical Oncology, University Hospital La Paz, Paseo de la Castellana 261, 28046 Madrid, Spain; zamorapilar@gmail.com; 9 Department of Cardiology, University Hospital La Paz, Paseo de la Castellana 261, 28046 Madrid, Spain; teresa.lopez@salud.madrid.org

**Keywords:** anthracyclines, cardiotoxicity, epirubicin, breast cancer, adverse drug reaction, *POLRMT*, heart

## Abstract

Anthracyclines are among the most used chemotherapeutic agents in breast cancer (BC). However their use is hampered by anthracycline-induced cardiotoxicity (AIC). The currently known clinical and genetic risk factors do not fully explain the observed inter-individual variability and only have a limited ability to predict which patients are more likely to develop this severe toxicity. To identify novel predictive genes, we conducted a two-stage genome-wide association study in epirubicin-treated BC patients. In the discovery phase, we genotyped over 700,000 single nucleotide variants in a cohort of 227 patients. The most interesting finding was rs62134260, located 4kb upstream of *POLRMT* (OR = 5.76, P = 2.23 × 10^−5^). We replicated this association in a validation cohort of 123 patients (P = 0.021). This variant regulates the expression of *POLRMT*, a gene that encodes a mitochondrial DNA-directed RNA polymerase, responsible for mitochondrial gene expression. Individuals harbouring the risk allele had a decreased expression of *POLRMT* in heart tissue that may cause an impaired capacity to maintain a healthy mitochondrial population in cardiomyocytes under stressful conditions, as is treatment with epirubicin. This finding suggests a novel molecular mechanism involved in the development of AIC and may improve our ability to predict patients who are at risk.

## 1. Introduction

Anthracyclines, especially doxorubicin (DOX) and epirubicin (EPI), are very effective and widely used chemotherapeutic drugs, alone or in combination regimens [1,2,3]. They are important agents in both (neo-)adjuvant and metastatic breast cancer (BC) treatment [4,5,6,7]. A recent meta-analysis performed by the Early Breast Cancer Trialists’ Collaborative Group (EBCTCG) reported a decrease in mortality by 20–30% [4]. Nevertheless, the duration of anthracycline based-therapy is limited by the risk for cumulative cardiac toxicity due to their affinity for myocardial tissue [8], which has led to the implementation of a maximum cumulative amount of drug that can be used for treatment [9,10,11].

Anthracycline-induced cardiotoxicity (AIC) appears in 8–75% of cancer survivors [3], depending on the cumulative dose, and ranges from a reversible drop in the left ventricular ejection fraction (LVEF) to heart failure and even cardiac death [12,13]. Severe AIC occurs in up to 8.7% of patients treated with high doses of anthracyclines [14,15]. In contrast, mild AIC leads to an increase in morbidity and mortality of 72% [12,16]. AIC is so important that it surpasses relapse and metastasis as the leading cause of death among BC survivors older than 66 years old who have survived more than five years after initial diagnosis [6,17]. Remarkably, among the group of anthracyclines, EPI presents a lower toxicity than its stereoisomers, because it has different pharmacokinetic characteristics that allow the use of up to twice the cumulative dose [5,18,19]. Despite the fact that EPI is considered to be a safer option, it has a lower therapeutic effect and sometimes requires higher doses [3,5,19], which eventually leads to a similar risk/benefit ratio as for DOX [3].

Currently, the exact underlying mechanism of AIC still remains unclear [20], although several hypotheses have been put forward, such as the generation of mitochondrial reactive oxygen species (ROS), disruption of mitochondrial biogenesis, and the induction of ferroptosis [20]. Aside from the cumulative dose, there are some widely known clinical risk factors that play an important role in the development of AIC, i.e., age, gender, hypertension, diabetes and smoking [21]. Nevertheless, these are not sufficient factors in order to explain all inter-individual variability observed [7,13,22,23].

The involvement of a genetic component has been proposed, and several studies have identified genetic variants that may explain these differences among patients and shed some light on the causes of AIC [24]. However, in particular in BC patients, very few genetic associations have been found and subsequently replicated.

Variant *CBR3* V244M (rs1056892) was first reported by Volkan-Salanci et al. [25], suggesting a harmful effect; subsequently, this association has been studied multiple times [26,27,28,29]. This common missense variant is located in the carbonyl reductase 3 gene involved in the formation of toxic secondary alcohol metabolites of anthracyclines [30,31]. rs1056892 affects expression of *CBR3*, which seems to correlate with the risk of AIC in paediatric patients [28,29] and BC patients [25,26,27]. The involvement of this variant in AIC seems to be irrefutable, and in vivo experiments support its effect [30,31].

Schneider et al. [32] identified and replicated an intergenic variant (rs28714259) related to susceptibility to developing anthracycline-induced congestive heart failure. The authors demonstrated that this variant was involved in long-range regulation of a glucocorticoid receptor known to play an essential role in the correct development of the fetal heart, as well as in the maintenance of the adult heart in different animal models [32].

The single nucleotide variant (SNV) rs7542939 reported by Wells et al. [33] is a variant close to the *PRDM2* gene, which is a tumour suppressor gene that encodes a zinc finger protein; it also plays a critical role in the repair of DNA double-strand breaks mediated by *BRCA1*, and works as a regulator of an oxidative stress protection gene. Its impairment exacerbates AIC in mouse models [33], and rs7542939 regulates the expression of *PRDM2* in some tissues.

Ruiz-Pinto et al. [11] identified and replicated the missense variant rs7933877 in *ETFB*, encoding a subunit of the mitochondrial flavoprotein beta, involved in the catabolism of fatty acids and amino acids via electron transfer to the electron transport chain. It has been shown in rat models that doxorubicin exerts a downregulation of this gene, which subsequently resulted in a decreased energy production, especially in heart, where the oxidation of fatty acids is the main energy source [11].

Unfortunately, these genetic variants are not sufficient to allow an accurate stratification of patients based on their individual genetic risk to develop AIC. Furthermore, the studies listed above were most often performed in DOX-treated individuals. Nowadays, EPI is becoming the preferred option for treatment amongst anthracyclines. However, no genetic studies have been published yet with regard to this particular drug.

Therefore, to identify novel genetic variants associated with AIC, we conducted a two-stage genome-wide association study (GWAS) in epirubicin-treated BC patients and addressed the functional significance of our findings.

## 2. Materials and Methods

### 2.1. Patients

A total of 227 female BC patients (137 cases and 90 controls) who visited the Leuven Multidisciplinary Breast Cancer Centre (University Hospital Leuven, Leuven, Belgium) between 2000 and 2010 were included in the discovery cohort [34]. The replication cohort consisted of 123 female BC patients (59 cases and 64 controls), enrolled in Hospital Universitario La Paz (Madrid, Spain) were analysed [21].

Cases and controls from both cohorts were treated with EPI and had normal cardiac function prior to the chemotherapy treatment. For all of them, echocardiographic evaluations before (baseline) and after treatment were available. Information regarding age at diagnosis, gender, primary tumour type, cumulative dose (mg/m^2^), location of tumour, use of radiotherapy, cardiovascular history and annotations on cardiovascular events, was obtained from medical records.

In order to ensure the homogeneity of the cohort and that the cardiac events were treatment-related, all patients with an age at diagnosis over 75, or with a baseline left ventricular ejection fraction (LVEF) less than 55% or any kind of cardiac pathology prior to treatment were excluded from the study.

Based on the literature [35,36,37] and the counsel of specialists in cardiology, patients were classified as follows: (1) Controls, if they had a decrease in LVEF of less than 10% compared to the baseline value, and had a final endpoint of more than 53 points. All controls included in the study received at least 1 cycle of EPI and were followed up for more than 12 months. (2) Mild cases, if they presented with a decrease in LVEF of more than 10% with a lowest LVEF over 53%. (3) Severe cases, if the decrease in LVEF was greater than 10% and the lowest LVEF was less than 53%, and/or they presented with any degree of symptomatic heart failure.

### 2.2. Genotyping and Quality Control

DNA samples of discovery and replication cohorts were quantified using Invitrogen^TM^ Quant-iT^TM^ Picogreen^TM^ dsDNA Reagent (Invitrogen by Thermo Fisher Scientific, Life Technologies Corporation, Eugene, OR, USA) and DTX 800 Multimode Detector (Beckman Coulter Inc, Fullerton, CA, USA), and genotyped using the Infinium Global Screening Array-24 v2.0 Beadchip (Illumina, San Diego, CA, USA) following the manufacturers’ recommended protocols. This array allows interrogating 759,993 markers across the genome. Genotyping quality assessment was performed using Illumina GenomeStudio v2.0.4 (Illumina, San Diego, CA, USA).

Quality filters, involving the exclusion of all samples with a rate of missing genotype data higher than 5% and the exclusion of all markers with a call rate lower than 0.95, were carried out with PLINK (v1.90b) [38,39]. Principal component analysis [40] was performed using the packages “scales” and “snpStats” for R (v3.6.3) [41], including samples from European (CEU), Iberian (IBS), Southern Han Chinese (CHS) and Yoruba (YRI) populations; outliers were excluded from subsequent analysis.

### 2.3. Data Imputation

Data imputation was performed using Minimac4 of the Michigan Imputation Server [42] with the HRC 1.1 reference panel. The genotype calling threshold was set to 0.7. Detailed inspection of the region of interest was carried out using LocusZoom [43] (http://locuszoom.org/, accessed on 21 December 2020). Region of interest was defined as the region that is limited by the farthest SNV with an *r*^2^ > 0.3 at each side of the associated variant.

### 2.4. Statistical Analysis

In order to identify clinical factors associated with AIC development, statistical analysis of the clinical variables was conducted independently for both the discovery and replication cohort by Student’s *t*-test using SPSS software (version 19, IBS Corp., New York, NY, USA); *p*-values < 0.05 were considered significant.

We identified associations with individual SNVs using logistic regression analysis in the discovery cohort by comparing severe and mild cases with controls; in addition, we performed an extreme phenotype analysis, comparing exclusively the severe cases with controls [44]. Both analyses were performed using PLINK [38,39] and included all significant clinical covariables previously identified. The genomic inflation factor (λ) was also calculated using PLINK [38,39].

The quantile-quantile (Q-Q) plot comparing the distribution of observed *p*-values with the expected ones, and the Manhattan-type plot were plotted using the “qqman” package of R [41]. All SNVs that reached a significance of less than 5 × 10^−5^ in the logistic regression analysis of the discovery phase were selected for further validation in the replication phase.

### 2.5. Functional annotation

To assess the potential functional impact of the identified variants we used Phenoscanner [45,46] Ensembl [47], ENCODE project [48,49,50,51], NIH Roadmap Epigenomics project (http://www.roadmapepigenomics.org/, accessed on 19 August 2021), the Genotype-Tissue Expression (GTEx) portal (gtexportal.org), and the University of California Santa Cruz (UCSC) Genome Browser [52].

## 3. Results

The clinical characteristics of both cohorts are shown in Table 1. Higher cumulative doses were associated with higher risk of developing AIC in both cohorts and therefore cumulative dose was included as a covariable in the subsequent logistic regression analyses.

**Figure 1:**
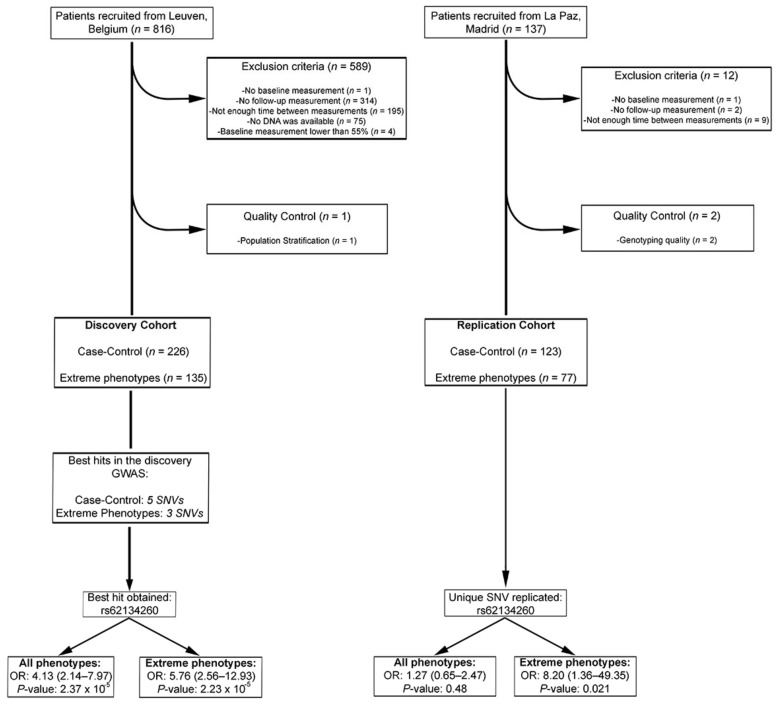
Flowchart of the patients included in the study. All *p*-values and Odds Ratios (ORs) given are from logistic regression analyses including the cumulative dose of epirubicin as a covariate.

After quality control, a total of 226 out of 227 BC patients of the discovery cohort and 725,785 SNVs were included in subsequent analyses (Figure 1). One sample was discarded due to stratification (Figure S1).

**Figure 2:**
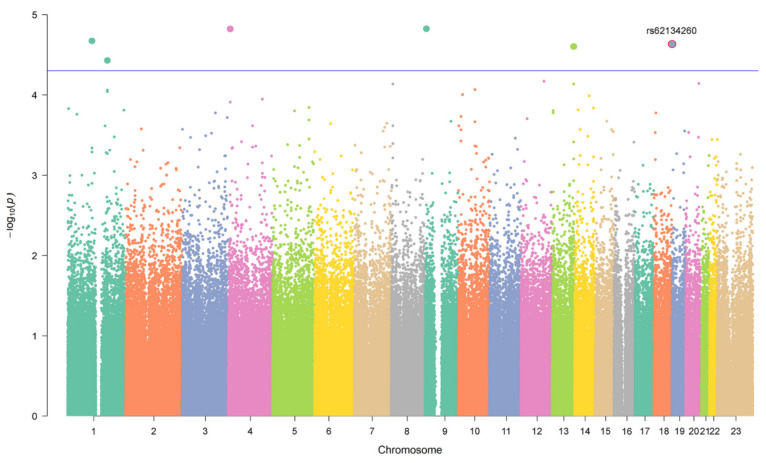
Manhattan Plot of the genome-wide association analysis in the discovery cohort for the case-control analysis. The association between the genotypes of 725,785 single nucleotide variants and risk of developing anthracycline-induced cardiotoxicity is shown. For the analysis, the cumulative epirubicin dose was used as covariate. All replicated SNVs are encapsulated in a red circle, as well as its name is displayed. The blue line represents the *p*-value threshold of 5 × 10^−5^.

Single SNV association analysis in the discovery phase identified six SNVs that reached a *p*-value < 5 × 10^−5^ (Figure 2 and Figure S2). Three of them are located in known genes, rs377189 (an intronic variant in *RCL1*), rs2270271 (a 3′ UTR variant in *GPR78*) and rs66539320 (an intronic variant located in a long non-coding RNA gene in chromo-some 1), whereas the other three are intergenic SNVs close to different genes: rs11185202 (located at 74 kb from the 5′ of *AMY1C*, and at 136 kb from the 3′ of *AMY1B*), rs62134260 (located at 4 kb from the 5′ of *POLRMT*, and at 2 kb from the 5′ of *FGF22*), and rs8000668 (located at 98 kb from the 5′ end of *ARHGEF7*, and at 101 kb from the 5′ end of *ANKRD10*).

Subsequently, we repeated the analysis excluding all mild cases (46 severe cases and 90 controls). Three additional SNVs rs382092 (an intronic variant located in a long non-coding RNA gene in chromosome 1), rs17687727 (a non-coding transcript exon variant located in a long non-coding RNA gene in chromosome 2), and rs6099854 (located at 225 kb from the 3′ of *PMEPA1*, and at 214 kb from the 3′ of *C20 orf85*) were identified, together with rs62134260 identified in the first analysis (Table 2).

For the replication phase, associations with AIC for these nine candidate variants were assessed by logistic regression analysis (Table 2) in the replication cohort. Only the intergenic SNV rs62134260 identified in both analyses, case control and extreme-phenotype approaches, was found to be significantly associated with AIC in the replication cohort when extreme phenotypes were considered (OR: 8.2 [1.36–49.35]; *p*-value = 0.021; OR _combined_: 4.0 [2.2–7.3], *p*-value = 7.10 × 10^−6^).

**Figure 3:**
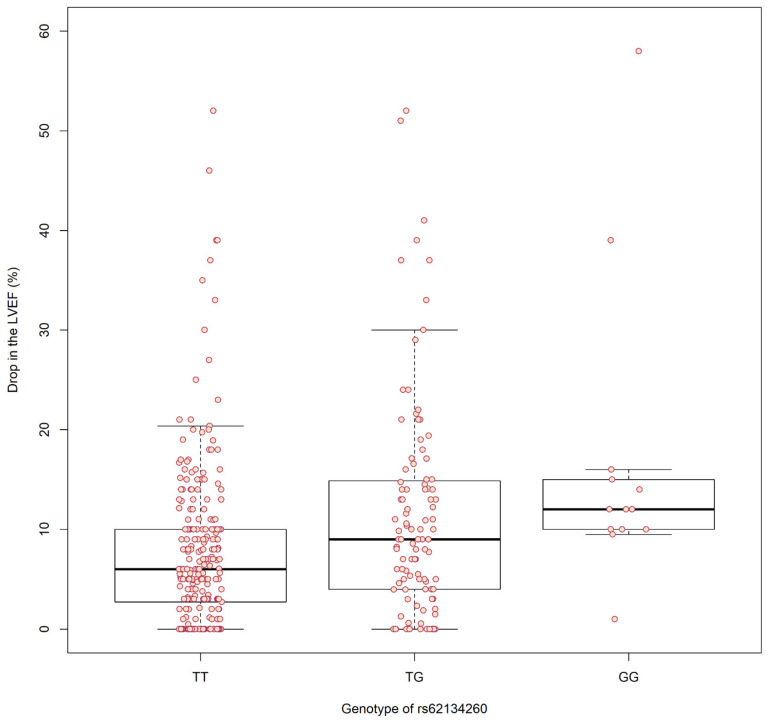
Boxplot representing the drops in left ventricular ejection fraction (LVEF) (%) of each patient according to their rs62134260 genotype. Patients are indicated by red circles.

Moreover, linear regression analysis of rs62134260 revealed a significant association between the genotype and the drop in LVEF (Discovery beta = 5.08 [2.71–7.44], *p*-value = 3.7 × 10^−5^. Combined beta = 3.50 [1.76–5.25], *p*-value = 1.02 × 10^−4^). Graphical depiction of this association can be seen in Figure 3.

**Figure 4:**
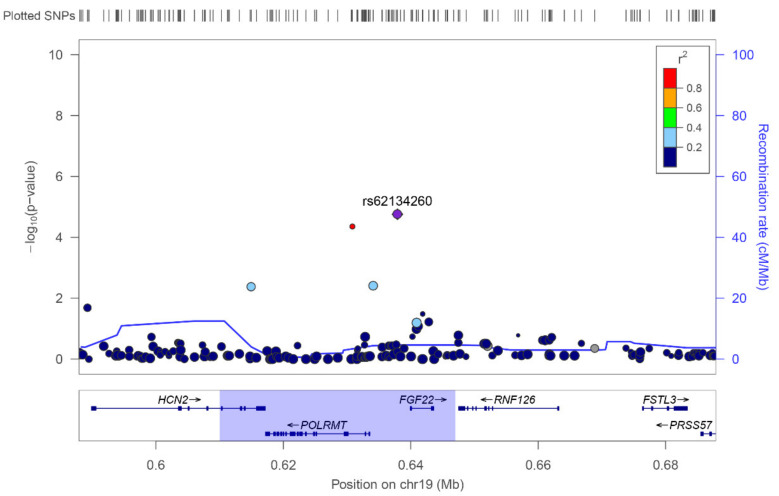
Genetic landscape surrounding rs62134260, plotted using LocusZoom (http://locuszoom.org/, accessed on 14 September 2021) with imputed data of the extreme phenotypes analysis (discovery cohort). Each dot represents a genetic variant; on the *y*-axis the statistical significance (−*log*_10_(*p*-value)) is represented, and on the *x*-axis the chromosomal position. The colour of the dots indicates the linkage disequilibrium value (*r*^2^) of the given SNV with rs62134260. The size of the dot reflects the square-root of the sample size (sample size may be affected by imputation quality of each sample). rs62134260 is indicated by a purple diamond. Blue highlighted region delimits the region with *r*^2^ > 0.3 regarding rs62134260.

In order to fine-map the rs62134260 association, we subsequently imputed 120 additional variants into the region of interest of the discovery cohort and tested their associations with AIC (Figure 4). We identified a new significant signal at rs11669897 (OR: 4.52 [2.02–8.10], *p*-value = 3.39 × 10^−5^). However, it showed weaker evidence of association, and the significance disappeared after adjustment for rs62134260, confirming a single association signal (Figure 4). This was expected, given the linkage disequilibrium present between both SNVs (0.83 in the European population).

The genes closest to rs62134260 are *POLRMT*, *FGF22* and *RNF126* (Figure 4). *FGF22* is located at 2 kb downstream and encodes a fibroblast growth factor mainly expressed in skin and brain. *POLRMT* is the second closest gene, located 4 kb upstream, and encodes the mitochondrial DNA-directed RNA polymerase. *RNF126* is located at 10 kb and encodes an ubiquitin ligase that targets proteins for degradation.

As our replicated variant is located in an intergenic region, we expected it to be linked to regulatory functions rather than affecting the function of proteins encoded by surrounding genes. Therefore, we further explored its regulatory effect. For this purpose, we explored its overlap with the histone marks of active promoters and active enhancers (H3K9ac, H3K4me1, and H3K27ac) using ENCODE data via UCSG and NIH Roadmap Epigenomics project data, showing that SNV rs62134260 overlapped with an H3K4me1 mark (often found near regulatory elements), and also with a strong enhancer in a B-lymphocyte ENCODE cell line (Figure S3). Additionally, this genomic region contains potential binding sites for key transcription factors (PKNOX1, PBX3, CEBPB) (Figure S3). Furthermore, we also performed eQTL analyses using data from GTEx Portal identifying rs62134260 as a robust cis-expression quantitative trait locus (cis-eQTL) in heart tissue (Heart—Atrial Appendage *p*-value = 4.2 × 10^−6^ and Heart—Left Ventricle *p*-value = 7.1 × 10^−5^), showing that the G allele decreases the expression of *POLRMT* in this tissue. No eQTLs that modify the expression of *FGF22* or *RNF126* were found. In conclusion, rs62134260 appears to be involved in the risk to develop AIC through modulation of the expression of *POLRMT*, encoding the mitochondrial DNA-directed RNA polymerase. This gene, whose product is required for mitochondrial gene expression and for providing the primers for the initiation of replication of the mitochondrial genome, is thus as a strong candidate for being involved in the susceptibility to AIC.

Lastly, we explored in both cohorts the associated variants described in the literature, however none of them showed a significant association (Table S1).

## 4. Discussion

The inter-individual variability in AIC susceptibility remains largely unexplained and there was an urgent need to identify valuable predictive markers for risk stratification, and to elucidate the precise molecular mechanisms underlying this severe adverse drug reaction. Several genetic variants have been associated with AIC, mainly in DOX-treated patients. Nevertheless, most of them have not been properly replicated [53,54,55]. This can be explained by the heterogeneity of the study designs, in which patients of different ages with different cancer types and/or treated with different anthracyclines were combined. To our knowledge, our study is the most homogeneous study reported thus far, as it includes exclusively BC patients treated with EPI in both discovery and replication cohorts in order to minimise heterogeneity and increase the chance of identifying true signals of association.

We identified and replicated a novel SNV rs62134260 associated with risk of developing AIC. Our findings indicate that this variant affects *POLRMT* mRNA expression and that dysregulation of the expression of this gene is a potential molecular mechanism underlying this risk. *POLRMT* is the only mitochondrial RNA-polymerase and is therefore a key component of mitochondrial gene expression and mitochondrial replication [56]. *POLRMT* is an essential gene, since knock-out (KO) mice are not viable due to embryonic lethality and conditional KO mice died after six weeks due to dilated cardiomyopathy [57]. Heterozygous mice showed a drastically decreased *POLRMT* protein level in heart, skeletal muscle, and liver [57], indicating the high demand for this protein in those tissues. It was also noted that a reduction of gene dosage does not prompt any kind of aberrant phenotype, since heterozygous mice seem to be viable, fertile and healthy [57]. It is well known that compounds that cause some kind of inhibition of the normal functioning of *POLRMT*, either in vivo or in vitro, create a significant decrease in the abundance of the mtDNA population, a reduction of the mitochondrial protein synthesis rate, and decreased mitochondrial respiration [58]. All these effects lead to a decrease in mitochondrial activity, and hence cause a decrease in adenosine triphosphate (ATP) production [58].

Cardiomyocytes require large amounts of ATP, due to the continuous energy-demanding contractions [59]. To maintain constant ATP production, cardiomyocytes rely on a great number of mitochondria. Malfunctioning mitochondria are continuously being replaced by processes involving mitophagy, replication and biogenesis [59]. The importance of mitochondria in the cardiomyocyte is illustrated by the fact that they occupy up to 35% of the total cell volume [60,61] and generate 90% of the ATP the cardiomyocyte consumes [62]. Moreover, any kind of interference with the replication process results in a gradual reduction of the mitochondrial population that eventually will lead to cardiotoxicity [59]. This became evident [63] when evidence started accumulating that doxorubicin-induced mitochondrial dysfunction plays a major role in cardiomyocyte death [64,65,66]. Moreover, it has been shown that anthracyclines directly interfere with the normal functioning of mitochondria in cardiac cells [4].

Bearing the above in mind, we propose that the decreased expression of *POLRMT* in risk allele carriers does not provoke any kind of phenotype by itself; however, when the cardiomyocyte is exposed to anthracyclines and a higher rate of mitochondrial renovation becomes critical, the decreased levels of mitochondrial RNA-polymerase are not sufficient to meet the increased demand. This inability to maintain a sufficient amount of healthy mitochondria will result in a substantial lack of ATP and the liberation and oxidation of iron, leading to cell death due to either energy depletion or ferroptosis. At the phenotypic level, the depletion of cardiomyocytes may eventually cause a decrease in the LVEF, cardiac problems, or even cardiac death. Our hypothesis explains the observed differences in sensitivity among patients treated with EPI. Nevertheless, functional studies in isogenic Human induced Pluripotent Stem Cells-Cardiomyocytes such as cell viability, ROS generation, superoxide generation, and mitochondrial integrity, are required in order to demonstrate the functional impact of the risk allele on the expression of *POLRMT*, and the role of the encoded mitochondrial RNA-polymerase in cardiomyocyte damage due to epirubicin. Whereas our finding is promising, our study has some limitations due to the retrospective design and the relatively small sample size. To prevent the discovery of spurious associations due to lack of statistical power, we conducted a two-stage study, including a replication step. This provides an important and independent statistical confirmation, and strongly decreases the probability of identifying associations by chance. Nonetheless, additional replication in a large prospective cohort with a longer follow-up after treatment would be desirable.

We also assessed in our patients four genetic variants already described to show a robust association with cardiotoxicity risk in DOX-treated breast cancer patients. We were not able to replicate these variants in our cohort of EPI-treated breast cancer cases. This may be due to the fact that this anthracycline has different pharmacokinetics than its stereoisomer DOX.

We believe that our results may shed some new light on the biological mechanisms underlying the risk of developing AIC in these patients. Nevertheless, functional studies to demonstrate how the presence of the risk allele G contributes to the increased damage in cardiomyocytes in the presence of EPI, as well as replication in a prospective cohort, are still required.

## Figures and Tables

**Table 1:** Analyses of the main clinical characteristics of discovery and replication cohorts.

Parameter	Discovery Cohort (*n* = 227)					Replication Cohort (*n* = 123)				
	Controls (*n* = 90)	Mild Cases (*n* = 92)	Severe Cases (*n* = 45)	*p*-Value (Case-Control)	*p*-Value (Extreme Phenotypes)	Controls (*n* = 64)	Mild Cases (*n* = 49)	Severe Cases (*n* = 10)	*p*-Value (Case-Control)	*p*-Value (Extreme Phenotypes)
Age (yr.) at diagnosis, median (IQR)	50 (45–55.75)	50 (44–57)	50 (45–56)	0.93	0.98	54 (43.75–60)	47 (40–53)	61.5 (47.25–67)	0.44	0.16
Cumulative dose (mg/m^2^), median (IQR)	300 (200–600)	600 (100–600)	600 (100–600)	**8.7** × **10^−6^**	**6.5** × **10^−4^**	511 (121–720)	540 (405–765)	486 (115–600)	0.92	**0.01**
Location of Tumor in Left Breast, No (%)	53 (58.89)	42 (46.15)	23 (50.00)	0.09	0.33	34 (53.13)	26 (53.06)	3 (30.00)	0.37	0.06
Use of Radiotherapy, No (%)	87 (9.67)	81 (88.04)	46 (100.00)	0.18	0.21	58 (90.63)	47 (95.92)	10 (100.00)	0.18	0.32
Radiotherapy treatment in Left Breast, No (%)	49 (54.44)	41 (44.57)	23 (51.11)	0.73	0.63	34 (53.13)	26 (53.06)	3 (30.00)	0.61	0.18
Bilateral Breast Cancer, No (%)	1 (1.11)	0 (0)	0 (0)	-	-	3 (4.69)	3 (6.12)	0 (0)	-	-

Abbreviations: IQR, Interquartile range. *p*-value in Case-Control analysis calculated using Student’s *t*-test for mild and severe cases against controls; *p*-value in Extreme Phenotypes analysis calculated using Student’s *t*-test for severe cases against controls. Significant *p*-values (*p*-val < 0.05) are highlighted in bold.

**Table 2:** Genetic variants identified in GWAS that achieved a significance of at least 5 × 10^−5^ in the logistic regression analysis.

CHR	SNV	Gene	Allele	AF	Analysis ^1^	Discovery *p*-Value	Cohort OR [95% CI]	Replication *p*-Value	Cohort OR [95% CI]
1	rs11185202	74 kb from *AMY1C*, 136 kb from *AMY1B*	T	0.41	Case-Control	2.13 × 10^−5^	0.36 [0.23–0.58]	0.88	0.96 [0.57–1.61]
1	rs66539320	lncRNA	G	0.18	Case-Control	3.72 × 10^−5^	0.33 [0.19–0.56]	0.56	1.24 [0.60–2.54]
1	rs382092	lncRNA	T	0.36	Extreme Phenotypes	2.32 × 10^−5^	4.03 [2.11–7.68]	0.31	0.57 [0.19–1.68]
2	rs17687727	lncRNA	A	0.16	Extreme Phenotypes	4.73 × 10^−5^	4.92 [2.29–10.66]	0.80	1.17 [0.34–4.07]
4	rs2270271	*GPR78*	T	0.46	Case-Control	1.51 × 10^−5^	0.38 [0.25–0.59]	0.84	0.95 [0.58–.’54]
9	rs377186	*RCL1*	A	0.44	Case-Control	1.50 × 10^−5^	0.35 [0.22–0.57]	0.38	0.81 [0.50–1.30]
13	rs8000668	98 kb from *ARHGEF7*, and 101 kb from *ANKRD10*	T	0.52	Case-Control	2.50 × 10^−5^	0.40 [0.26–0.62]	0.41	0.79 [0.46–1.38]
19	rs62134260	4 kb from *POLRMT*, and 2 kb from *FGF22*	G	0.16	Case-Control	2.37 × 10^−5^	4.13 [2.14–7.97]	0.48	1.27 [0.65–2.47]
					Extreme Phenotypes	2.23 × 10^−5^	5.76 [2.56–12.93]	**0.021**	8.2 [1.36–49.35]
20	rs6099854	225 kb from *PMEPA1*, and 214 kb from *C20orf85*	A	0.14	Extreme Phenotypes	3.77 × 10^−5^	6.57 [2.69–16.09]	0.42	0.42 [0.05–3.47]

^1^ Case-Control (Controls against Mild and Severe Cases, Discovery Cohort *N* = 226, Replication Cohort *N* = 123), Extreme Phenotypes (Controls against Severe Cases, Discovery Cohort *N* = 135, Replication Cohort *N* = 77). Abbreviations: AF, Allele Frequency in European Population; OR, Odds Ratio; CI, Confidence Interval; *N*, Sample Size; Kb, Kilobases; CHR, Chromosome; SNV, Single-nucleotide variant. Cumulative dose is used as covariate in each analysis. Significant *p*-values in the replication cohort (*p*-value < 0.05) are highlighted in bold.

## Data Availability

The data presented in this study are available upon request.

## Supplementary Materials

The following are available online at https://www.mdpi.com/article/10.3390/pharmaceutics13111942/s1, Figure S1: Principal Component Analysis (PCA) of genetic data for the 227 samples from the discovery cohort (green dots) for the 725,785 SNVs that passed quality control, including CEU (black dots), IBS (dark blue dots), CHS (red dots) and YRI (light blue dots) populations from the 1000 Genomes project (https://www.internationalgenome.org/, accessed on 6 July 2016). Principal components 1 and 2 were plotted. The sample indicated by a red arrow was excluded from further analysis. Figure S2: Quantile-Quantile plot showing the distribution of observed −log_10_(*p*-values) from logistic regression analysis of the 725,785 SNVs from the discovery cohort plotted against expected −log_10_(*p*-values). PLINK-estimated genomic inflation factor (λ) = 1, indicating no apparent population stratification. Figure S3: Regulatory landscape of the rs62134260 obtained from ENCODE and NIH Roadmap Epigenomics project data via UCSG Genome Browser. The histone modification tracks, the transcription factor ChIP-seq clusters and the integrated regulation of transcription are displayed. Red vertical line represent the exact SNV position. Table S1: Previously described variants and their association to Anthracycline Induced Cardiotoxicity in our cohorts via logistic regression analysis.
