# Supplementary material for: POLRMT as a Novel Susceptibility Gene for Cardiotoxicity in Epirubicin Treatment of Breast Cancer Patients
Source: Pharmaceutics. 2021 Nov 16;13(11):1942. doi: 10.3390/pharmaceutics13111942 (PMC8622627; doi:10.3390/pharmaceutics13111942)
Supplement: Supplementary file 1 [file pharmaceutics-13-01942-s001.zip › pharmaceutics-1444154-supplementary.pdf]

pharmaceutics

Supplementary Materials: *POLRMT* as a Novel Susceptibility Gene for Cardiotoxicity in Epirubicin Treatment of Breast Cancer Patients

Alejandro Velasco-Ruiz, Rocio Nuñez-Torres, Guillermo Pita, Hans Wildiers, Diether Lambrechts, Sigrid Hatse, Danielle Delombaerde, Thomas Van Brussel, M. Rosario Alonso, Nuria Alvarez, Belen Herraez, Christof Vulsteke, Pilar Zamora, Teresa Lopez-Fernandez and Anna Gonzalez-Neira

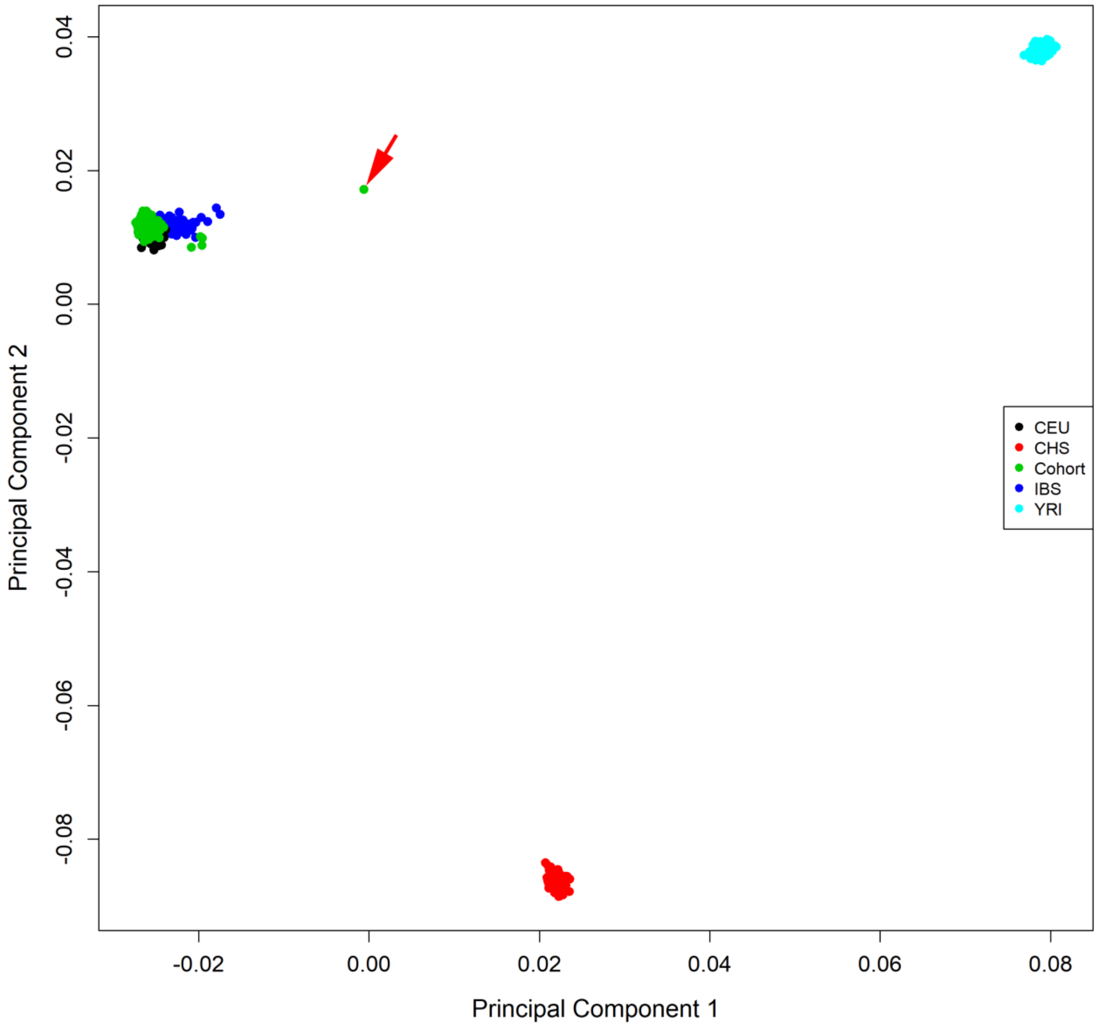

Figure S1. Principal Component Analysis (PCA) of genetic data for the 227 samples from the discovery cohort (green dots) for the 725,785 SNVs that passed quality control, including CEU (black dots), IBS (dark blue dots), CHS (red dots) and YRI (light blue dots) populations from the 1000 Genomes project (https://www.internationalgenome.org/, access on 06 July 2016). Principal components 1 and 2 were plotted. The sample indicated by a red arrow was excluded from further analysis.

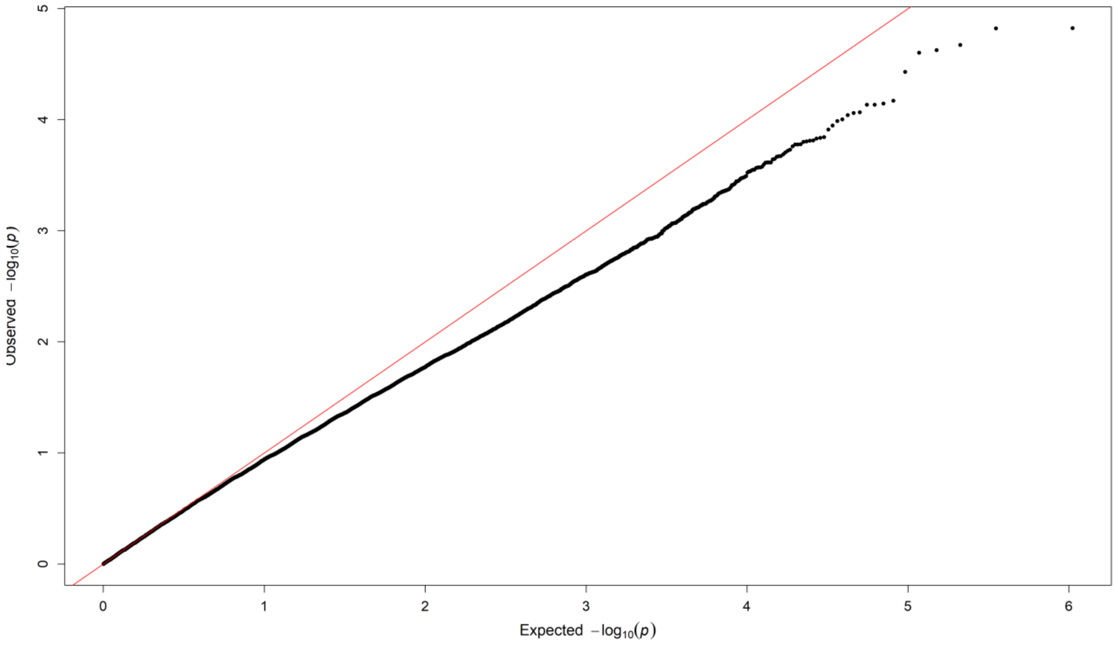

Figure S2. Quantile-Quantile plot showing the distribution of observed –log10(*p*-values) from logistic regression analysis of the 725,785 SNVs from the discovery cohort plotted against expected –log10(*p*-values). PLINK-estimated genomic inflation factor (λ) = 1, indicating no apparent population stratification.

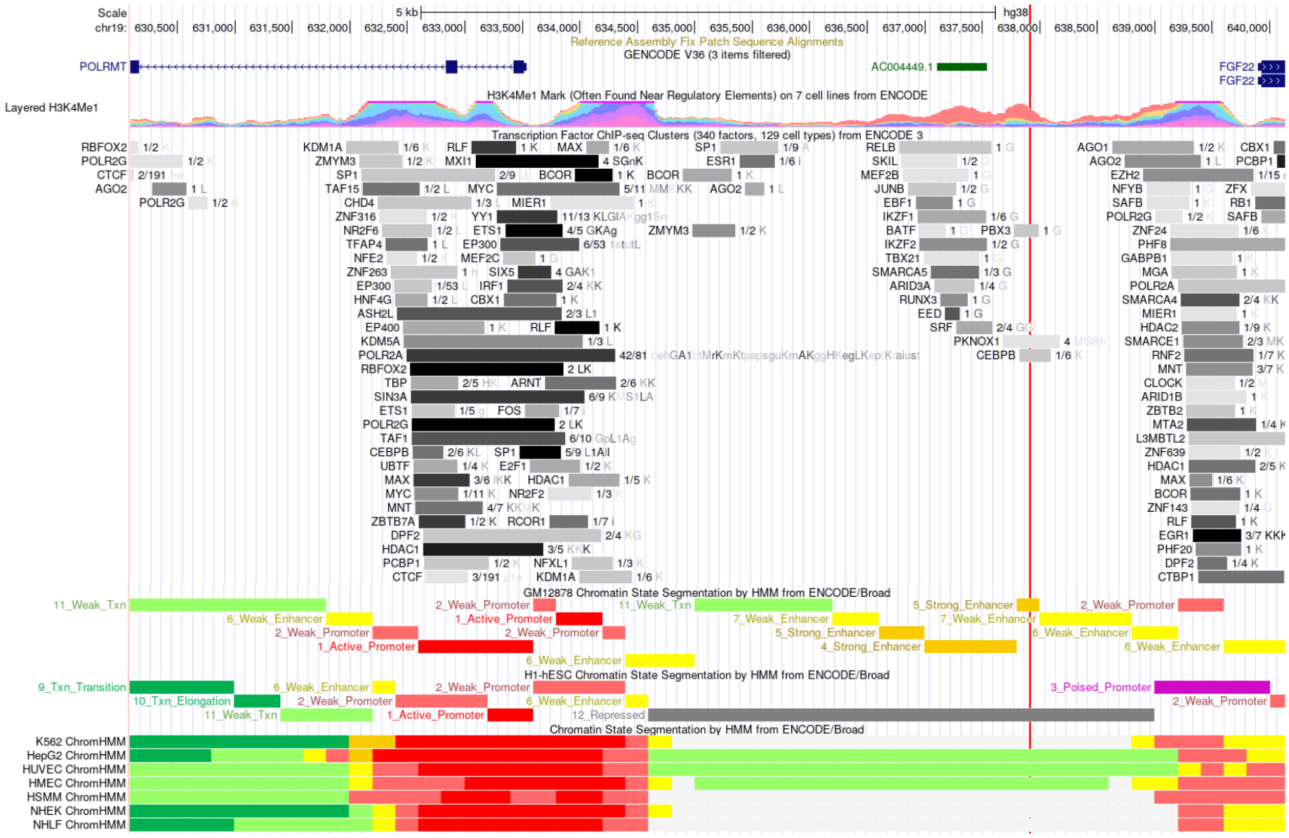

Figure S3. Regulatory landscape of rs62134260 obtained from ENCODE and NIH Roadmap Epigenomics project data via UCSG Genome Browser. The histone modification tracks, the transcription factor ChIP-seq clusters and the integrated regulation of transcription are displayed. Red vertical line indicates the exact SNV position.

Table S1. Previously described variants and their association to Anthracycline-Induced Cardiotoxicity in our cohorts via logistic regression analysis.

SNV	Gene	Discovery Cohort				Replication Cohort			
		Case-Control [1]		Extreme Phenotypes [1]		Case-Control [1]		Extreme Phenotypes [1]	
		***p*-Value**	**OR**	***p*-Value**	**OR**	***p*-Value**	**OR**	***p*-Value**	**OR**
rs1056892	CBR3	0.659	0.91	0.599	0.86	0.160	1.49	0.706	0.779
rs7542939	PRMD2	0.882	0.96	0.716	0.87	0.186	0.59	0.303	0.33
rs28714259	Intergenic	0.953	0.98	0.700	1.17	0.311	0.63	0.515	1.62
rs79338777	ETFB	0.149	0.53	0.404	0.63	0.355	1.51	0.628	1.47

[1] Case-Control (Controls against Mild and Severe Cases, Discovery Cohort *N* = 226, Replication Cohort *N* = 123), Extreme Phenotypes (Controls against Severe Cases, Discovery Cohort *N* = 135, Replication Cohort *N* = 77), Abbreviations: OR, Odds Ratio; *N*, Sample Size. Cumulative dose is used as covariate in each analysis. Significant *p*-values (*p*-val < 0.05) are highlighted in bold.
